# Editorial: Non-invasive brain stimulation in psychiatric disorders: From bench to bedside

**DOI:** 10.3389/fpsyt.2022.1106558

**Published:** 2023-01-16

**Authors:** Chih-Sung Liang, Po-Han Chou, Shao-Cheng Wang, Alexander T. Sack, Kuan-Pin Su

**Affiliations:** ^1^Department of Psychiatry, Beitou Branch, Tri-Service General Hospital, National Defense Medical Center, Taipei, Taiwan; ^2^Department of Psychiatry, National Defense Medical Center, Taipei, Taiwan; ^3^Department of Psychiatry, China Medical University Hsinchu Hospital, China Medical University, Hsinchu, Taiwan; ^4^Department of Psychiatry, Taoyuan General Hospital, Ministry of Health and Welfare, Taoyuan, Taiwan; ^5^Department of Mental Health, Johns Hopkins Bloomberg School of Public Health, Baltimore, MD, United States; ^6^Section Brain Stimulation and Cognition, Department of Cognitive Neuroscience, Faculty of Psychology and Neuroscience, Maastricht University, Maastricht, Netherlands; ^7^Maastricht Brain Imaging Centre (MBIC), Maastricht, Netherlands; ^8^Centre for Integrative Neuroscience (CIN), Maastricht University, Maastricht, Netherlands; ^9^Department of Psychiatry and Neuropsychology, School for Mental Health and Neuroscience (MHeNs), Brain + Nerve Centre, Maastricht University Medical Centre+ (MUMC+), Maastricht, Netherlands; ^10^College of Medicine, China Medical University, Taichung, Taiwan; ^11^Mind-Body Interface Laboratory (MBI-Lab), China Medical University and Hospital, Taichung, Taiwan; ^12^An-Nan Hospital, China Medical University, Tainan, Taiwan

**Keywords:** noninvasive brain stimulation, transcranial magnetic stimulation, transcranial direct current stimulation, non-regional specificity, rTMS, tDCS

The development of effective treatment modality for psychiatric disorders is an enduring goal of translational research and evidence-based medicine. In recent decades, progress in neuroscience has identified the dysfunctional brain circuits and networks that may underpin the pathogenesis of psychiatric disorders (1). Non-invasive brain stimulation (NIBS) is a set of techniques that can modulate the excitability of large-scale networks in the brain (2). Studies have shown promising results in circuit-based psychiatric treatments in either diagnosis- or symptom-based clinical conditions (3–5).

The current Special Issue, *Non-invasive brain stimulation in psychiatric disorders: From bench to bedside*, in Frontiers in Psychiatry, is dedicated to collect high-quality studies that explore the possible mechanisms for the therapeutic effects of NIBS, including molecular, genetics, neuroimaging, and neurophysiological aspects. The relevance for application of transcranial magnetic stimulation (TMS) in treating psychiatric disorders is driven by the development of new protocols and sequences (2). The Food and Drug Administration agency of the United States approved rTMS as a treatment for medication-resistant patients with MDD in 2008 (6). The therapeutic effects of repetitive transcranial magnetic stimulation (rTMS) were also observed in other psychiatric conditions, including MDD (Harika-Germaneau et al.; Spitz et al.), suicidal ideation (Huang et al.), smoking cessation (Chen et al.), and methamphetamine use disorder (Mikellides et al.). Unlike TMS, TES uses low intensity currents to modulate the excitability of targeted networks in the brain. TES is an umbrella term for a variety of different stimulation modalities, such as transcranial direct current stimulation (tDCS) and transcranial alternating current stimulation. Evidence supports TES as a therapeutic tool in depression (Chang et al.), attention-deficit/hyperactivity disorder (Sobral et al.), and social cognition in schizophrenia (Kannen et al.). These findings from clinical trials and practical experiences suggest that one of the strength of NIBS may lie in its non-regional specificity.

The circuit-based neuromodulation of NIBS may explain the heterogeneity of psychiatric disorders than can be treated with TMS/TES (Figure 1). For example, high-frequency rTMS over left dorsolateral prefrontal cortex (DLPFC) or low-frequency rTMS over right DLPFC are usually applied in the treatment of MDD (Yamada et al.); however, targeting other brain regions also revealed therapeutic effects for MDD, such as ventromedial prefrontal cortex (PFC), orbitofrontal cortex, and ventrolateral PFC (7). The magnetic stimuli applied may regulate the activity of local circuits in the interneurons including fibers projecting to other distant brain regions, which depend on the intrinsic properties and geometrical orientation of the fibers within the stimulated brain region (8). The interconnection between networks of the brain may thus also explain the non-regional specificity of NIBS effects for the same psychiatric disorder.

**Figure 1:**
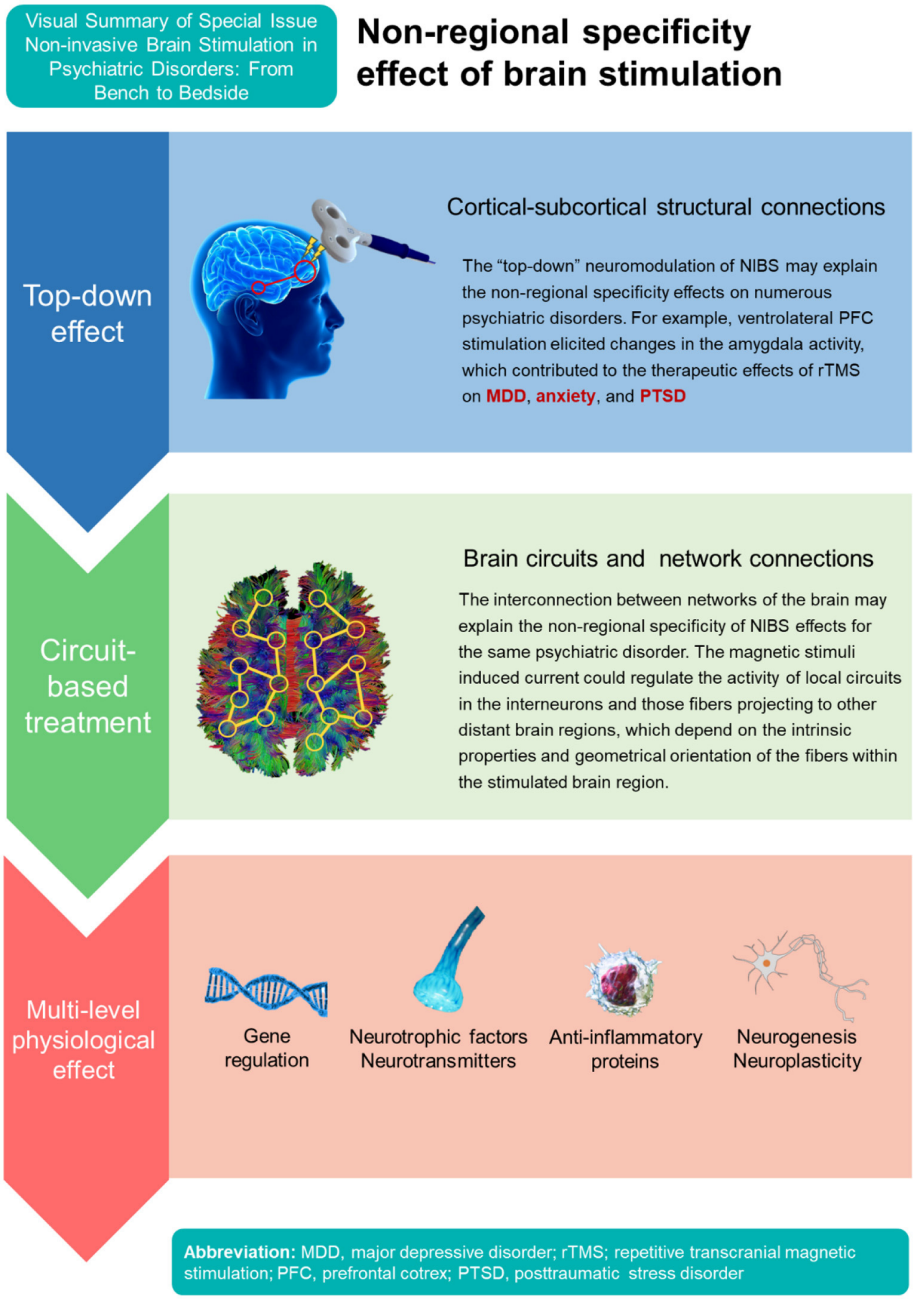
Mechanisms of non-regional specificity effect of non-invasive brain stimulation.

In addition, stimulating left DLPFC showed therapeutic effects not only for MDD but also in obsessive-compulsive disorder (9), suicidal ideation (Huang et al.), and methamphetamine use disorder (Mikellides et al.). The neuromodulation can be considered a “top-down” intervention, working at the level of brain networks and then affecting neurogenesis, neuroplasticity, and neurocircuitry (8, 10). For example, a recent study using TMS applied to the ventrolateral PFC elicited changes in the amygdala activity (11). The amygdala processes valenced stimuli, influences emotion, and contributes to a wide array of behavioral and brain disorders (12). Therefore, the top-down neuromodulation of TMS on the amygdala may enable a specific brain region stimulation for the treatment of numerous psychiatric disorders showing aberrant activity in the amygdala, such as MDD, anxiety, and posttraumatic stress disorder (11). Importantly, the therapeutic mechanisms of rTMS also involve neurotransmitter systems (e.g., serotonin, dopamine), neurotrophic factors, anti-inflammatory protein, and various molecular pathways (e.g., extracellular signal-regulated kinase 1/2, endocannabinoid systems) (8, 13, 14). Therefore, the cortical-subcortical structural and functional connections as well as various gene/protein expression and pharmacological modulation may all support the non-regional specificity of NIBS effects for various psychiatric disorders.

Take genetic molecular mechanisms for example, preliminary evidence suggests that the neurobiological effects of gene activation/regulation, *de novo* protein expression, synaptic morphological changes, homeostatic processes and glial function might underlies the long-term after effects of NIBS (15). Althoufh the effects of rTMS may produce long-term therapeutic effects on various psychiatric disorders (15), evidence suggests that rTMS pattern, intensity, frequency, train duration, intertrain interval, intersession interval, pulse and session number, pulse width, and pulse shape can alter motor excitability, long term potentiation-like facilitation, and the clinical antidepressant response (16). The response of rTMS varied widely among depressed patients. A study including 1,132 participants reported that around a half of patients could not achieve treatment response after rTMS treatment (Caulfield and Brown). Therefore, exploration of treatment predictors could help guide the choice of NIBS protocols that are more effective in precision medicine. A naturalistic observational study found that early improvement of depression can be a useful predictor for treatment response for rTMS treatment (Harika-Germaneau et al.). Another study examined clinical and neuroimaging biomarkers of treatment response with rTMS among treatment-resistant depression (6). The reported predictors included depression type, gender, depression severity, and the average volume of the left part of the superior frontal and the caudal middle frontal regions (6).

Advances in psychiatric practice lie in translating evidence from bench to beside. A better understanding of the neurobiological mechanism of NIBS has become an important piece in modern psychiatric practice. The non-region specificity of NIBS provides a window into circuit-based treatment for numerous psychiatric disorders. We believe the findings of the Special Issue could inspire future research to improve psychiatric treatment with precision NIBS applications.

## Author contributions

All authors listed have made a substantial, direct, and intellectual contribution to the work and approved it for publication.
